# Deep learning-based feature selection for detection of autism spectrum disorder

**DOI:** 10.3389/frai.2025.1594372

**Published:** 2025-06-25

**Authors:** Ibrahim Nafisah, Nermine Mahmoud, Ahmed A. Ewees, Mohamed G. Khattap, Abdelghani Dahou, Safar M. Alghamdi, Ibrahim A. Fares, Mohammed Azmi Al-Betar, Mohamed Abd Elaziz

**Affiliations:** ^1^Department of Statistics and Operations Research, College of Sciences, King Saud University, Riyadh, Saudi Arabia; ^2^Faculty of Human Science, Galala University, Suez, Egypt; ^3^Department of Computer, Damietta University, Damietta, Egypt; ^4^Technology of Radiology and Medical Imaging Program, Faculty of Applied Health Sciences Technology, Galala University, Suez, Egypt; ^5^School of Computer Science and Technology, Zhejiang Normal University, Jinhua, China; ^6^Department of Mathematics and Statistics, College of Science, Taif University, Taif, Saudi Arabia; ^7^Department of Mathematics, Faculty of Science, Zagazig University, Zagazig, Egypt; ^8^Artificial Intelligence Research Center (AIRC), College of Engineering and Information Technology, Ajman University, Ajman, United Arab Emirates; ^9^Hourani Center for Applied Scientific Research, Al-Ahliyya Amman University, Amman, Jordan; ^10^Faculty of Computer Science and Engineering, Galala University, Suez, Egypt

**Keywords:** autism detection, deep learning, resting-state functional MRI (rs-fMRI), feature selection, Hiking Optimization Algorithm, dynamic-opposite learning, double attractors

## Abstract

**Introduction:**

Autism Spectrum Disorder (ASD) is a neurodevelopmental condition characterized by challenges in communication, social interactions, and repetitive behaviors. The heterogeneity of symptoms across individuals complicates diagnosis. Neuroimaging techniques, particularly resting-state functional MRI (rs-fMRI), have shown potential for identifying neural signatures of ASD, though challenges such as high dimensionality, noise, and small sample sizes hinder their clinical application.

**Methods:**

This study proposes a novel approach for ASD detection utilizing deep learning and advanced feature selection techniques. A hybrid model combining Stacked Sparse Denoising Autoencoder (SSDAE) and Multi-Layer Perceptron (MLP) is employed to extract relevant features from rs-fMRI data in the ABIDE I dataset, which was preprocessed using the CPAC pipeline. Feature selection is enhanced through an optimized Hiking Optimization Algorithm (HOA) that integrates DynamicOpposites Learning (DOL) and Double Attractors to improve convergence toward the optimal subset of features.

**Results:**

The proposed model is evaluated using multiple ASD datasets. The performance metrics include an average accuracy of 0.735, sensitivity of 0.765, and specificity of 0.752, surpassing the results of existing state-of-the-art methods.

**Discussion:**

The findings demonstrate the effectiveness of the hybrid deep learning approach for ASD detection. The enhanced feature selection process, coupled with the hybrid model, addresses limitations in current neuroimaging analyses and offers a promising direction for more accurate and clinically applicable ASD detection models.

## 1 Introduction

Autism can be defined as a set of behavioral manifestations that include restricted activities, barriers to communication, as well as social interaction problems. A more accurate term for this condition is autism spectrum disorder (ASD) (Lord et al., 2018). Diagnosis can be made as early as 18 to 24 months, when symptoms become distinguishable from typical development and other cognitive or developmental challenges (Sayers et al., 2023). ASD is classified under neurodevelopmental disorders in the Diagnostic and Statistical Manual of Mental Disorders (DSM-5) and is associated with language impairments, poor social engagement, and limited or repetitive interests and activities. Parents of children with ASD face considerable psychological, physical, and financial burdens (John and Sala, 2018). Various tools have been used to diagnose ASD, such as the Autism Spectrum Quotient (AQ), the Childhood Autism Rating Scale (CARS-2), and the Screening Tool for Autism in Toddlers and Young Children (STAT) (Al-Hendawi et al., 2023). These assessments help identify symptoms and determine the severity of the condition, facilitating early intervention and support. However, there is a pressing need for more advanced and accurate methods, particularly those employing artificial intelligence (AI) to enhance the effectiveness of these traditional techniques.

According to the Global Burden of Disease (GBD) Study, ASD ranks among the six most common developmental disabilities in children under 5 years old. The prevalence of ASD has increased significantly in recent decades, likely due to greater awareness and improved recognition of the condition (Zeidan et al., 2022). In 2010, approximately 52 million children were diagnosed with ASD, translating to a prevalence of 7.6 per 1,000 individuals. In 2018, the Centers for Disease Control and Prevention (CDC) reported that 1 in 59 children had ASD, a figure that rose to 1 in 44 by 2020 (Zeidan et al., 2022). Studies in Europe and the United States suggest that ASD diagnoses have increased markedly over the last two decades, from 0.48% to 3.13% (Zeidan et al., 2022). However, most research on ASD prevalence in Arab countries has focused on wealthier nations. A systematic meta-analysis found that ASD prevalence rates vary across Oman, the UAE, Saudi Arabia, Bahrain, Kuwait, and Qatar (Sayers et al., 2023). In Egypt, ASD prevalence estimates have varied significantly, ranging from 5.4 per 1,000 to as high as 33.6% (Zeidan et al., 2022). However, Egyptian studies are often limited to specific regions, institutional settings, and small sample sizes.

Diagnosing ASD remains challenging due to its frequent co-occurrence with other disorders such as epilepsy, attention deficit hyperactivity disorder (ADHD), and sensory processing disorders, often resulting in delayed or missed diagnoses (Simonoff et al., 2008). Recent research estimates that 1 in 36 children in the United States are diagnosed with ASD, a figure substantially higher than in previous decades, attributable to broadened diagnostic criteria and increased public awareness (Christensen, 2016; Qin et al., 2024). This growing prevalence places considerable strain on healthcare systems, with families often incurring annual therapy and treatment costs exceeding 60, 000 per child, underscoring the critical need for accessible and advanced detection tools (Lavelle et al., 2014; Huda et al., 2024).

Traditional ASD diagnosis primarily relies on parent-reported developmental milestones and behavioral observations, which are inherently subjective and susceptible to cultural and gender biases (Patil et al., 2024; Bahathiq et al., 2022). Consequently, many adolescents and adults, especially females without intellectual disabilities, remain undiagnosed until secondary mental health issues arise (Giarelli et al., 2010). In response, neuroimaging techniques such as resting-state functional MRI (rs-fMRI) have gained prominence by revealing abnormal connectivity patterns within brain networks related to social cognition and sensory processing (Supekar et al., 2013). Yet, bringing such findings to the clinical realm necessitates surmounting the computational challenges: one rs-fMRI dataset comprises tens of thousands of regional connectivity features but scarcely over 1,000 subjects even in public databases like the Autism Brain Imaging Data Exchange (ABIDE) (Di Martino et al., 2014). Therefore, there is an urgent imperative to develop effective diagnostic methods for ASD, which not only facilitate early intervention but also play a crucial role in managing the condition's global prevalence. Implementing such diagnostic tools can provide timely support and resources, ultimately improving outcomes for individuals with ASD and their families.

Machine learning (ML) offers a promising solution by detecting subtle neural signatures associated with ASD. Nonetheless, high dimensionality and noise in neuroimaging data continue to challenge model accuracy (Mellema et al., 2022; Fares et al., 2025). Feature selection (FS) techniques, such as recursive feature elimination, have become essential for removing redundant connections while preserving biomarkers related to social attention and executive function (Mellema et al., 2022; Bahathiq et al., 2022; Fares and Abd Elaziz, 2025). Hybrid approaches that combine deep learning (DL) with FS have demonstrated notable success. For example, methods integrating the Adaptive Bacterial Foraging (ABF) algorithm with Support Vector Machine Recursive Feature Elimination (SVM-RFE) have shown high performance in ASD detection (Lamani and Benadit, 2023). Similarly, convolutional neural networks (CNNs) hybridized with Elephant Herding Optimization (EHO) algorithms have been applied to multiple fMRI datasets, yielding promising results in identifying ASD patients (Chola Raja and Kannimuthu, 2023).

However, despite such progress, various challenges remain. The biological heterogeneity of ASD implies that no neural marker is universally applicable; connectivity changes differ between toddlers and adults, between verbal and non-verbal individuals, or between those with and without genetic syndromes (Alzubaidi et al., 2023). Reproducibility is also constrained by small sample sizes and heterogeneous preprocessing pipelines at imaging sites (Schielen et al., 2024; Chola Raja and Kannimuthu, 2023).

This paper aims to propose a modified version of the autism detection model based on the strengths of DL and FS techniques. In general, the DL model combining a Stacked Sparse Denoising Autoencoder (SSDAE) and a Multi-Layer Perceptron (MLP) is used to extract the relevant features (Liu et al., 2024). Following by using an enhanced version of Hiking Optimization Algorithm (HOA) as an FS technique (Oladejo et al., 2024). This enhancement is conducted through using Dynamic Opposites Learning (DOL) (Ahmad et al., 2022) and Double Attractors (He and Lu, 2022) to enhance the convergence toward the optimal subset of relevant features. These approaches have been established in their performance in different applications. For example, DOL has applied engineering problems (Cao et al., 2023; Xu et al., 2020), job shop scheduling (Yang et al., 2022), IIR system identification (Niu Y. et al., 2022), and skin cancer detection (Dahou et al., 2023). Has applied to enhance design of structures (Kaveh and Yousefpoor, 2024), color image compression (Yao et al., 2025), and KELM diabetes classification (Zhu et al., 2025).

The contributions of this study can be stated as follows:

- Development of an autism detection approach using DL and an enhanced FS model based on a modified version of the HOA algorithm.
- Integration of SSDAE and MLP for learning feature representations from rs-fMRI data and performing feature extraction.
- Introducing a modified version of HOA using the dynamic opposite-based learning and double attractors.
- Evaluation of the performance of the developed autism detection technique on multiple datasets and comparison with other well-known methods.

The organization of this paper is given as follows: Section 2 introduces the related works of using different AI models to detect autism. Section 3 presents the basic information of the Hiking Optimization Algorithm (HOA), Dynamic-Opposite Learning (DOL), and Double Attractors. The stages of the proposed autism detection model are presented in section 4. The experimental results and discussion are introduced in Section 5. Finally, the conclusion and future works are presented in Section 6.

## 2 Related works

The development of AI-based models for detecting ASD has seen notable progress, particularly with the integration of FS techniques and DL algorithms. Over the past decade, various studies have explored innovative methods for leveraging neuroimaging data, such as rs-fMRI, in combination with DL approaches. This section will review the most influential works that have contributed to the development of ASD detection models, highlighting key advancements in both AI methodologies and neuroimaging techniques used in ASD diagnosis.

Earlier work of Di Martino et al. (2014) and Guan and Liu (2021) described Autism Brain Imaging Data Exchange (ABIDE), a multi-site rs-fMRI repository, allowing for high-dimensional analysis of functional connectivity in ASD, and driving development in ML techniques. Nielsen et al. (2013) displayed multisite fMRI classification with SVMs, but with high-dimensional and site-related biases limiting it. The DL transition began with graph-based techniques. Parisot et al. (2018) started with graph convolutional networks (GCNs) representing functional connectivity in brain graphs, with 70% accuracy in ABIDE through encoding non-linear relationships between regions of interest (ROIs). In parallel, Heinsfeld et al. (2018) utilized convolutional neural networks (CNNs) for raw fMRI time-series, with renewed emphasis on automatization of feature extraction in an effort to reduce manual ROI selection. Hybrid architectures soon dominated: Eslami et al. (2019) combined SVM-RFE FS with 3D CNNs, and 88% accuracy in ABIDE through isolating discriminative connections in the default mode network (DMN). Similarly, Wang et al. (2020) designed a multi-atlas feature ensemble scheme and showed FS preceding training with DL aided generalizability improvement over ABIDE sites.

FS techniques specific to neuroimaging data gained prominence. Niu X. et al. (2022) optimized site-wise feature reproducibility with LASSO regularization, while Abraham et al. (2017) proposed a deep embedded feature selection (DEFS) algorithm, training FS layers and autoencoders together, and discovering cerebellar and somatosensory connectivity to be significant biomarkers. For multi-modal data, Abbas et al. (2023) merged structural MRI and fMRI features with attention, mapping 87% accuracy for ABIDE-II. Graph-methods saw a quantum jump with Li et al. (2020), utilizing graph neural networks (GNNs) for investigating modular connectivity profiles, mapping 80% accuracy, and indicating thalamocortical impairment in ASD.

Further, hybrid meta-heuristic algorithms along with CNNs achieved 98.6% on the ABIDE dataset (Chola Raja and Kannimuthu, 2023). Likewise, DL models like YOLOv8 while performing the analysis on facial images, showed 89.64% classification accuracy with a F1-score of 89% (Gautam et al., 2023). The proposed adaptive bacterial foraging optimization along with SVM-RFE and mRMR and followed by the graph convolutional network classifier obtained an accuracy of 97.512% (Lamani and Benadit, 2023).

Liu et al. (2024) introduced MADE-for-ASD, which integrates the power of various brain atlases and demographic information with fMRI. It presented an accuracy as high as 96.40% by highlighting the essential ASD-relevant brain regions. Furthermore, this efficient model is extendable and available openly for public adoption. A meta-analysis in Ding et al. (2024) emphasized the classification performance of these deep-learning models in ascertaining the disorder amongst children; thereby it may be exploited in extending present diagnostic methodologies. Chen et al. (2024) introduced DeepASD, an adversary-regularized GNN, aligning feature distributions between modalities (fMRI + SNPs), and mapping state-of-the-art 93% AUC-ROC performance for ABIDE-II. While the study Joe (2024) proposed using AI robots integrated with visual strategies to enhance social and communication skills in children with ASD. The author employed interactive robots to deliver structured visual stimuli and personalized learning experiences to improve engagement and skill retention. The author used a tuned CNN model, and it achieved an accuracy of 96% in the detection of ASD.

The study of Khan and Katarya (2025) proposes a new scheme, WS-BiTM, fusing White Shark Optimization (WSO) for FS and Bidirectional Long Short-Term Memory (Bi-LSTM) for classification for ASD improvement. WSO is utilized for selecting significant features out of sets of datasets for autism screening, and then these are processed with Bi-LSTM for efficient sequential processing. WSO-Bi-LSTM overcomes overfitting and computational efficiency issues effectively. Baseline algorithms outdo through comparative studies with 97.6%, 96.2%, and 96.4% accuracy for datasets for toddlers, adults, and kids, respectively, and proving its efficiency as a dependable tool for ASD classification.

The contribution of Abu-Doleh et al. (2025) introduces a two-step model for improving ASD classification with volumetric brain MRI images. First, subcortical structures are extracted and processed with a 3D autoencoder in order to detect regions of interest for analysis for ASD-related analysis. Secondly, these regions are classified with a Siamese Convolutional Neural Network (SCNN). SCNN achieved 66% accuracy with regions determined with the Mutual Information FS criterion. This contribution identifies the potential for fusing SCNNs and autoencoders for brain MRI-based ASD improvement. Jabbar et al. (2025) develop an ML model for early ASD screening by combining parent-reported questionnaires plus video analysis of child behavior. They achieved high accuracy through feature engineering by using data balancing techniques. Their hybrid algorithm outperforms traditional tools in AUC (0.92). The solution enables low-cost, mobile-friendly screening, particularly beneficial in resource-limited settings where clinical access is restricted.

## 3 Background

Optimization algorithms play a crucial role in enhancing the effectiveness of AI models by improving FS processes and model performance. Among these, the Hiking Optimization Algorithm (HOA) has shown promise as an effective tool for solving complex optimization problems, due to its human-inspired search mechanism that mirrors the dynamics of hiking. This section will introduce the key concepts behind HOA, Dynamic-Opposite Learning (DOL), and Double Attractors, which serve as the foundational techniques for the proposed autism detection model.

### 3.1 Hiking Optimization Algorithm

The Hiking Optimization Algorithm (HOA) is a metaheuristic inspired by hiking, where hikers navigate varying terrains to reach a peak (Oladejo et al., 2024). Similar to the unpredictable landscapes of hiking, optimization problems feature complex search spaces. HOA uses Tobler's Hiking Function (THF) to model hikers' movement, considering terrain elevation and distance. This approach mimics hikers' strategies of avoiding steep paths to maintain a steady pace, helping agents in HOA find optimal solutions while avoiding local optima. The algorithm's human-inspired structure makes it an efficient tool for solving complex optimization problems.

#### 3.1.1 Initialization

In the first step of the HOA, the initial positions of the hikers—analogous to search agents—are established randomly. This method ensures diversity in the search space, promoting a broad exploration of potential solutions. The position of each hiker, denoted as *X*_*i*_(*t*), is determined within a defined search space. This space is bounded by the upper limit *UB*_*i*_ and the lower limit *LB*_*i*_ for each dimension *j* of the decision variable. The initialization process follows the equation:


(1) $$X_{i}(t)=\left(UB_{i}-LB_{i}\right)\times \text{ rand}()+LB_{i}$$

where rand() is a uniformly distributed random variable within the range [0, 1].

#### 3.1.2 Modeling hiker speed using Tobler's Hiking Function

The next step is incorporating the widely recognized *Tobler's Hiking Function* (THF), a mathematical model formulated by the geographer Waldo Tobler. THF is an exponential function that estimates hikers' velocity based on the terrain's steepness. This function plays a crucial role in HOA, as it simulates the movement dynamics of search agents (hikers) within the optimization space.

The velocity of a hiker *i* at iteration *t*, denoted as $W_{i}\left(t\right)$, is computed using the following equation:


(2) $$\begin{array}{l} W_{i}\left(t\right)=6e^{-3.5|S_{i,t}+0.05|} \end{array}$$

where *S*_*i, t*_ represents the slope of the terrain at the hiker's position. The slope itself is determined by the elevation change (*dh*) and the distance traveled (*dx*), given by:


(3) $$\begin{array}{l} S_{i,t}=\frac{dh}{dx}=\tan\theta_{i,t} \end{array}$$

where θ_*i, t*_ is the inclination angle of the terrain, constrained within the range [0°, 50°].

The integration of THF into HOA ensures that the movement of hikers (agents) is guided by realistic terrain-based constraints. In essence, steeper inclinations result in slower movement speeds, mirroring real-world hiking behaviors. By leveraging this function, HOA dynamically adjusts the step sizes of agents in the optimization process, enhancing both exploration and exploitation capabilities.

#### 3.1.3 Exploitation phase

The exploitation phase of the HOA is responsible for refining the search process by guiding hikers (agents) toward promising regions in the optimization landscape. This phase leverages the social intelligence of hikers as a group and their individual cognitive abilities. A key parameter known as the sweep factor (SF) plays a crucial role in defining the balance between exploitation and exploration. The SF regulates the influence of the lead hiker on the movement of other hikers, controlling the extent of their deviation from the leader's trajectory. A higher SF value directs the HOA toward the exploitation phase, allowing agents to converge toward promising solutions. Conversely, a lower SF encourages exploration, enabling the algorithm to investigate diverse regions of the search space. The velocity of a hiker *i* at iteration *t* is updated by:


(4) $$\begin{array}{l} W_{i}\left(t+1\right)=W_{i}\left(t\right)+\gamma \times \left(X_{\text{best}}-\alpha_{i}\left(t\right)X_{i}\left(t\right)\right) \end{array}$$

where γ is a random number within the range [0, 1]. $W_{i}\left(t+1\right)$ and $W_{i}\left(t\right)$ represent the actual and initial velocities of the hiker, respectively. The variable *X*_best_ corresponds to the position of the lead hiker, representing the best solution found so far. Additionally, α_*i*_(*t*) denotes the sweep factor (SF), which takes values in the range [1, 3].

The updated position of hiker *i* at the next iteration, incorporating its velocity, is expressed as:


(5) $$\begin{array}{l} X_{i}\left(t+1\right)=X_{i}\left(t\right)+W_{i}\left(t\right) \end{array}$$

The complete implementation details of the Hiking Optimization Algorithm, including its initialization, velocity updates, and search mechanisms, are outlined in the pseudocode provided in Algorithm 1.

**Algorithm 1:**
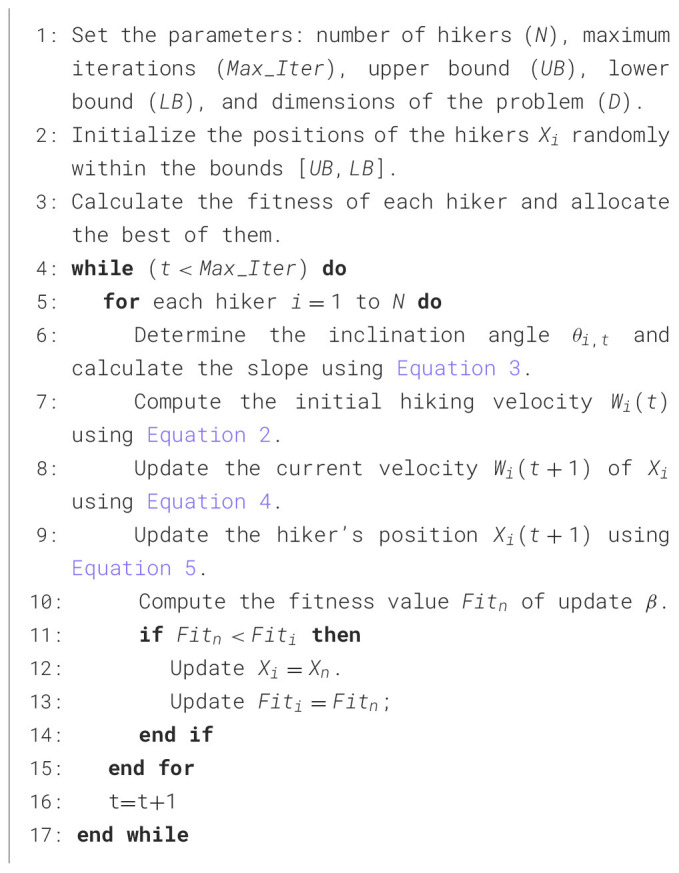
Pseudo-code of the HOA algorithm.

### 3.2 Dynamic-opposite learning

Metaheuristic optimization algorithms often struggle with premature convergence, leading to stagnation in local optima. Dynamic-Opposite Learning (DOL) is a recent strategy designed to enhance both exploration and exploitation by dynamically adjusting the search space (Xu et al., 2020). DOL builds upon Opposite-Based Learning (OBL) (Wang et al., 2011; Rahnamayan et al., 2007; El-Abd, 2011), which improves convergence by considering opposite solutions (Tizhoosh, 2005). Traditional OBL methods refine this concept but remain susceptible to local optima (Rahnamayan et al., 2007; Ergezer et al., 2009). To overcome this, DOL introduces an asymmetric and dynamically expanding search space, increasing population diversity and reducing stagnation. A random opposite number is used to create asymmetry, preventing premature convergence, while a weighting factor balances exploration and exploitation. By integrating DOL into metaheuristic frameworks, optimization performance is significantly enhanced, making it a powerful approach for solving complex problems.

The concept behind DOL is to expand the search space dynamically, rather than symmetrically, by introducing a random opposite number *X*^*RO*^, defined as:


(6) $$\begin{array}{l} X^{RO}=\text{ rand }\times X^{O},\text{ where rand }\in \left[0,1\right] \end{array}$$

(7) $$\begin{array}{l} X_{j}^{O}=a_{j}+b_{j}-X_{j},\;j=1,2,...,D \end{array}$$

where [*a, b*] represents the search domain of *X*. *D* is the dimension of *X*. In general, replacing the standard opposite number *X*^*O*^ with *X*^*RO*^ transforms the search into an asymmetric adaptive process, preventing premature convergence. A new candidate solution *X*^*DO*^ is then selected as:


(8) $$\begin{array}{l} X^{DO}=X+\text{ rand}\left(X^{RO}-X\right) \end{array}$$

To maintain feasibility, *X*^*DO*^ is adjusted if it falls outside the search boundaries [*a, b*]. However, as iterations progress, the search space may shrink, reducing the algorithm's exploitation capability. To counteract this, a weighting factor *w* is introduced, refining the final formulation:


(9) $$\begin{array}{l} X^{DOL}=X+w\times \left(X^{RO}-X\right) \end{array}$$

where *w* is a positive constant ensuring an optimal balance between exploration and exploitation.

#### 3.2.1 Dynamic opposite number

Let *X* be a real number in the search space, where *X*∈[*a, b*]. To introduce dynamic adaptation, the dynamic opposite number *X*^*DO*^ is defined as:


(10) $$\begin{array}{l} X^{DO}=X+w\times \text{ rand }\times \left(X^{O}-X\right), \end{array}$$

where *X*^*O*^ represents the opposite number of *X* as defined earlier in the OBL in Equation 7, *w* is a positive weighting factor controlling the expansion range, and *rand* is a random value sampled from (0, 1). The introduction of *w* ensures a balanced adaptation, preventing excessive search space contraction while enhancing exploration capabilities.

#### 3.2.2 Dynamic opposite point in multi-dimensional space

Extending the DOL approach to higher dimensions, consider *X* = (*X*_1_, *X*_2_, …, *X*_*D*_) as a point in a *D*-dimensional search space, where each coordinate *X*_*j*_ falls within the predefined range [*a*_*j*_, *b*_*j*_]. The opposite point in this space is denoted by $X_{j}^{O}$, as defined in Equation 7. The dynamic opposite point is formulated as:


(11) $$\begin{array}{l} X_{j}^{DO}=X_{j}+w\times \text{ rand }\times \left(\text{rand }\times X_{j}^{O}-X_{j}\right),\;j=1:D \end{array}$$

#### 3.2.3 DOL-based optimization

The proposed DOL strategy is applied iteratively to guide the optimization process. Given a population of candidate solutions *X*, each point undergoes transformation based on the dynamic opposite learning mechanism. The newly generated opposite candidates $X^{DO}=\left(X_{1}^{DO},X_{2}^{DO},\ldots ,X_{D}^{DO}\right)$ are assessed based on their objective function values.

The selection process follows a simple criterion:

If the fitness of *X*^*DO*^ surpasses that of *X*, the new candidate is accepted. Otherwise, *X*^*DO*^ is discarded, and the original *X* is retained.

To ensure boundary constraints are maintained, each $X_{j}^{DO}$ must satisfy:


(12) $$\begin{array}{l} X_{j}^{DO}\in \left[a_{j},b_{j}\right],\;j=1:D \end{array}$$

If any $X_{j}^{DO}$ falls outside this range, it is reinitialized as a random value within [*a*_*j*_, *b*_*j*_].

### 3.3 Double attractors

In this section, we introduce Double attractors as one of the most important operators that are used to enhance the balancing between exploration and exploitation (He and Lu, 2022). In general, the solutions in the metaheuristic algorithms are updated their values according to the shared information and personal knowledge. Moreover, the solutions during the updating process move toward the feasible solution that is considered as an attraction point. However, the process of balance between the main phases of MH techniques, named exploration and exploitation, is considered one of the main challenges that the MH algorithms suffer from them. Therefore, DA is used to handle this challenge, and this is achieved through using two attractors named *L*1 and *L*2.

Following (He and Lu, 2022), *L*1 is defined as in Equation 13.


(13) $$\begin{array}{l} L1_{ij}\left(t\right)=\phi_{1}\times p_{ij}\left(t\right)+\left(1-\phi_{1}\right)\times p_{bj}\left(t\right),i=1,2,...,N \end{array}$$

In Equation 13, *p*_*ij*_(*t*) indicates the historical best value at *j*th dimension of *X*_*i*_ the iteration *t*. Whereas *p*_*bj*_(*t*) indicates the value of *X*_*b*_ among dimension *j* among *t*th iteration. ϕ_1_ is a parameter that linearly decreased over iterations and it is defined.


(14) $$\begin{array}{l} \phi_{1}=\left(\beta_{2}-\beta_{1}\right)\times \frac{T-t}{T}+\beta_{1},\beta_{1},\beta_{2}=0.9 \end{array}$$

where *T* is the maximum number of iterations.

Moreover, the second attractor *L*2 is defined as:


(15) $$\begin{array}{l} L2_{ij}\left(t\right)=\phi_{2}\times p_{ij}\left(t\right)+\left(1-\phi_{1}\right)\times p_{bj}\left(t\right),i=1,2,...,N \end{array}$$

where ϕ_2_ denotes a constant parameter.

Finally, the solution can be updated using either *L*1 and *L*2 as defined in Equation 16.


(16) $$X_{ij}^{at}=\{\begin{array}{c} X_{ij}^{1}\;iff(X_{ij}^{1})<f(X_{ij}^{2})\\ X_{ij}^{2}\;otherwise \end{array}$$

(17) $$X_{ij}^{1}=\{\begin{array}{c} L1_{ij}+\beta \times |m_{j}-X_{ij}(t)|ln\frac{1}{u}\;if\;rand<0.5\\ L1_{ij}-\beta \times |m_{j}-X_{ij}(t)|ln\frac{1}{u}\;otherwise \end{array}$$

(18) $$X_{ij}^{2}=\{\begin{array}{c} L2_{ij}+\beta \times |m_{j}-X_{ij}(t)|ln\frac{1}{u}\;if\;rand<0.5\\ L2_{ij}-\beta \times |m_{j}-X_{ij}(t)|ln\frac{1}{u}\;otherwise \end{array}$$

## 4 Proposed method

Building on the foundational concepts of HOA, DOL, and Double Attractors introduced in the previous section, this section presents the developed autism detection model. The model integrates DL techniques with the modified HOA to enhance the diagnostic process by extracting meaningful features from raw data and optimizing FS for improved diagnostic accuracy.

### 4.1 Feature extraction process

The feature extraction process in the proposed framework involves leveraging multi-atlas fMRI data to identify discriminative features for ASD diagnosis following the proposed model and process in Liu et al. (2024). Functional connectivity matrices are derived from three brain atlases (AAL, CC, EZ) using Pearson correlation coefficients, which are flattened into one-dimensional vectors. These vectors are input into a Stacked Sparse Denoising Autoencoder (SSDAE) for pre-training, where sparsity and noise constraints are applied to learn robust feature representations. The SSDAE compresses the data into a reduced encoding, which is then fine-tuned using the MLP. The final 100-unit layer of the MLP extracts learned features, which are subsequently processed by an FS algorithm to identify the most relevant features for classification. This approach ensures the extraction of meaningful and discriminative features from multi-atlas fMRI data, optimizing the model for ASD diagnosis.

### 4.2 Feature selection process

The modified HOA plays a pivotal role in the FS process by refining the search for the most relevant features that contribute to accurate ASD detection. By integrating dynamic OBL, HOA enhances the ability to exploit known good solutions while avoiding local optima. The introduction of DA further improves the balance between exploration and exploitation, allowing the algorithm to explore diverse regions of the feature space and converge more effectively on the most discriminative features for ASD classification. This makes the HOA an essential tool for improving the diagnostic accuracy of the proposed model, as it ensures that only the most relevant and informative features are used for classification, which directly impacts the detection of ASD.

Followed by generating a set of solutions *X* and using its opposition using DOL as defined in Equation 11. Then determining the best *N* solutions from *X*∪*X*^*DOL*^ according to their fitness value. The next process is to update the solutions *X* using the operators of HOA and DA. This process of updating is conducted until the stop conditions are met. The details of the proposed model are given as follows.

#### 4.2.1 First stage

This stage aims to generate a suitable population *X* which has *N* solutions based on the DOL techniques. To achieve this task, the first step is to use Equation 19 to set the initial value for these solutions.


(19) $$\begin{array}{l} X_{i}=r_{5}\times \left(U_{j}-L_{j}\right)+L_{j},j=1,2,...,D,i=1,2,...,N \end{array}$$

where *D* denotes the dimension of *X* and *r*_5_∈[0, 1] refers to a random value. We apply Equation 11 to generate the opposite solutions *X*^*DO*^ for each *X*_*i*_, *i* = 1, 2, ..., *N*. Then we compute the fitness value for *X* and *X*^*DO*^, then select the best *N* solutions from *X*∪*X*^*DO*^ to form the initial solution *X*. In general, this step leads to enhancing the convergence rate toward the optimal solution.

#### 4.2.2 Second stage

This second stage aims to enhance the value of solutions *X* based on the operators of HOA and DA. This process is conducted by determining the best solution *X*_*b*_.

The next step is to determine the selected features using the current solution *X*_*i*_, and this is achieved by using the binary of *X*_*i*_ as defined in the following formula.


(20) $$BX_{ij}=\{\begin{array}{c} 1\;if\;r_{6}<0.5\\ 0\;otherwise \end{array}$$

In Equation 20, *r*_6_∈[0, 1] denotes a random value. After that, we evaluate the quality of the selected features which correspond to the ones in *BX*_*i*_, and this is performed through computing the fitness value (*Fit*_*i*_) as in the following equation.


(21) $$\begin{array}{l} Fit_{i}=\rho \times \gamma +\left(1-\rho \right)\times \left(\frac{\left|BX_{ij}\right|}{D}\right) \end{array}$$

In Equation 21, |*BX*_*ij*_| refers to the number of selected features. γ denotes the error of classification using the KNN classifier (we set *K* = 5). ρ∈[0, 1] refers to a parameter used to balance between the two terms of Equation 21.

The best *X*_*b*_ solution with the best *Fit* is then identified. Then we apply the operators of HOA as defined in Equations 2–5. Then we used the DA to enhance *X*, however, to reduce the time complexity of this stage, we used the following formula.


(22) $$X_{ij}=\{\begin{array}{c} X_{ij}^{at}\;if\;mod(t,20)==0\\ X_{ij}\;otherwise \end{array}$$

The steps of this stage are repeated until the stop conditions are met. Then the best solution is returned as the output of this stage.

#### 4.2.3 Third stage

**Figure 1:**
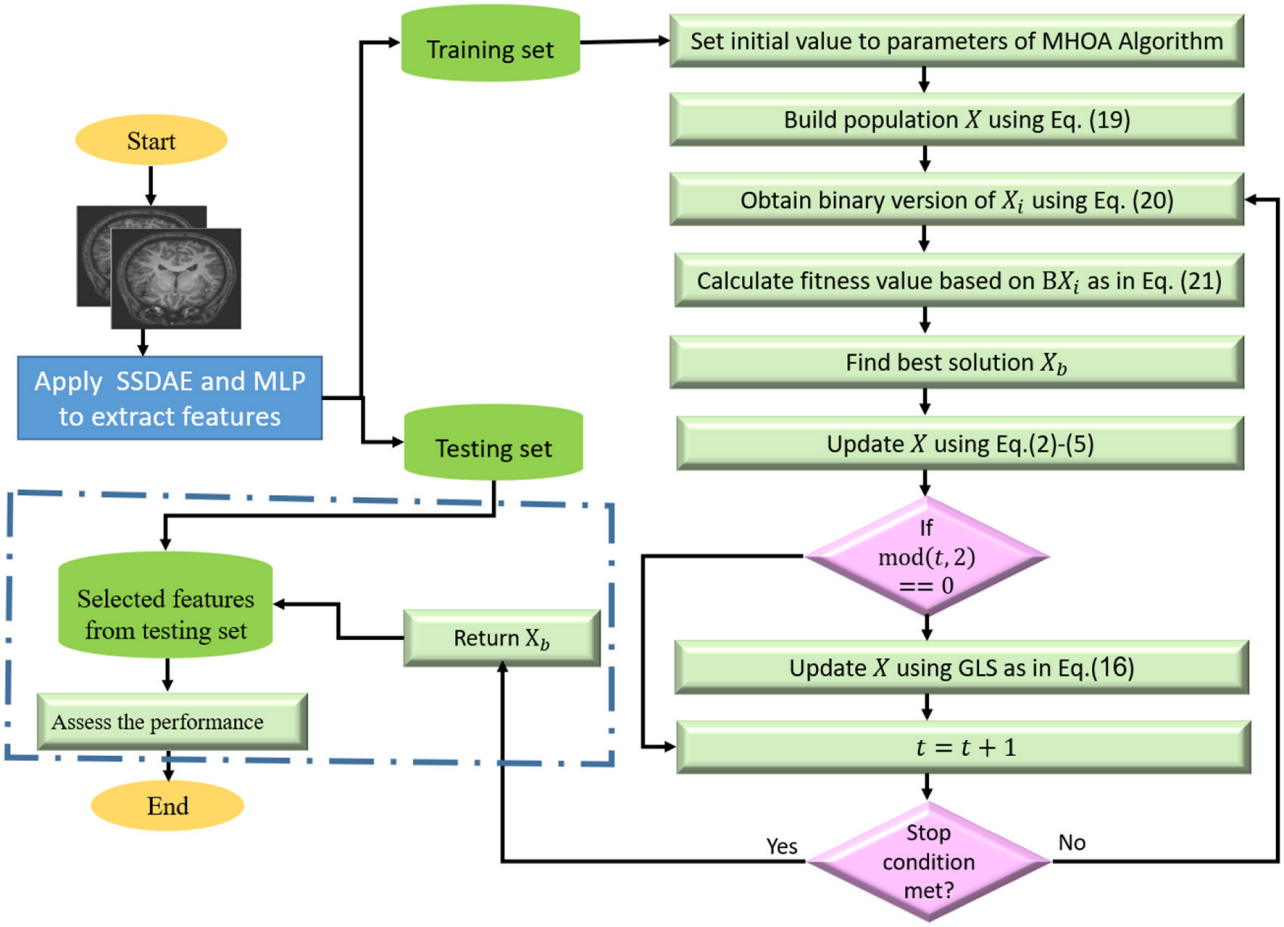
Phases of the developed autism detection based on the MHOA algorithm.

Finally, we used the testing set to assess the quality of the selected features, and this was conducted by generating the binary version of *X*_*b*_ is obtained using Equation 20. Then we select the relevant features from the testing set that correspond to the ones in *BX*_*b*_ and assess the quality of those features by computing the performance metrics of the predicted values obtained using the trained KNN model. The steps of the developed model are given in Figure 1.

## 5 Experimental results and discussion

Having outlined the architecture and processes of the proposed autism detection model, this section presents the results of the proposed autism detection model, including a comprehensive analysis of its performance. The effectiveness of the model is evaluated through various experiments, and its performance is compared with existing models to assess its strengths and limitations.

### 5.1 Dataset preparation

The rs-fMRI data used in this study were sourced from the ABIDE I dataset, comprising 505 autistic individuals and 530 typical controls (TCs). The dataset includes subjects with ASD and TC, with age ranges from 10.0 to 35.0 years for ASD and 10.0 to 33.7 years for TC across different sites. The data is preprocessed using the Configurable Pipeline for the Analysis of Connectomes (CPAC) pipeline, which includes essential steps such as slice timing correction, voxel intensity normalization, motion correction, nuisance signal removal, global signal regression, band-pass filtering, and spatial registration. After preprocessing and quality control, the final dataset consists of 1,035 samples distributed as follows: 623 samples for the training set, 308 samples for the validation set, and 104 samples for the test set. Functional connectivity matrices are generated using three brain atlases (AAL, CC, EZ), and the mean time series for each ROI is calculated following (Liu et al., 2024). These matrices are flattened into one-dimensional vectors, forming the input for the feature extraction model. Finally, we derived three datasets from ABIDE I based on the EZ, AAL, and CC atlases, designated as Dataset-1, Dataset-2, and Dataset-3, respectively.

### 5.2 Model configurations

The model architecture integrates an SSDAE followed by an MLP. For the SSDAE, pre-training was conducted with a learning rate of 0.001 using gradient descent (GD) and a batch size of 100. Sparsity and noise constraints were applied to enhance robust feature learning, with dropout set to 0.5 to reduce overfitting. The SSDAE comprises two autoencoder layers: the first encoding layer has 1,000 units, and the second reduces the representation to 600 units. The MLP was subsequently fine-tuned with a learning rate of 0.0005 using stochastic gradient descent (SGD) and a smaller batch size of 10 to allow for finer updates. Dropout was set to 0.3 for the MLP. The final layer of the MLP includes 100 units, outputting the learned features for each atlas. The number of training iterations was selected based on convergence behavior: the first SSDAE autoencoder was trained for 700 iterations, and the second for 1,000 iterations to ensure adequate reconstruction performance.

### 5.3 Evaluation metrics

The effectiveness of the suggested method, along with the performance of the comparison algorithms, is assessed using the following evaluation metrics:

- Accuracy:

(23) $$\begin{array}{l} \text{Accuracy}=\frac{TP+TN}{TP+TN+FP+FN} \end{array}$$

In this formula, *TP* and *TN* denote the true positive and true negative counts, while *FP* and *FN* represent the false positives and false negatives, respectively.

- Sensitivity:

(24) $$\begin{array}{l} \text{Sensitivity}=\frac{TP}{TP+FN} \end{array}$$

- Standard Deviation (StDev):

(25) $$\begin{array}{l} \text{StDev}=\sqrt{\frac{1}{N}\overset{N}{\underset{i=1}{\sum }}{\left(x_{i}-\bar{x}\right)}^{2}} \end{array}$$

Where, *N* refers to the total number of runs, *x*_*i*_ denotes the individual values, and $\bar{x}$ is the mean of those values.

### 5.4 Results and discussion

To assess the effectiveness of the proposed MHOA-based ASD detection model, experiments were conducted using multiple datasets derived from the ABIDE I database. Each dataset was preprocessed using a consistent pipeline to extract functional connectivity matrices, which were then transformed into feature vectors for analysis. The experiments used identical training and evaluation procedures across all algorithms to ensure a fair comparison.

The MHOA method was compared against six metaheuristic optimization algorithms: Hiking Optimization Algorithm (HOA), slime mold algorithm (SMA) (Ewees et al., 2023), Attraction-repulsion optimization algorithm (AROA) (Cymerys and Oszust, 2024), Harris hawk optimizer (HHO) (Abd Elaziz and Yousri, 2021), Great Wall Construction Algorithm (GWCA) (Guan et al., 2023), and gray wolf optimizer (GWO) (Helmi et al., 2021). These algorithms were selected based on their demonstrated success in FS and high-dimensional search spaces. All methods were evaluated using a k-nearest neighbors (KNN) classifier with consistent parameter settings. Evaluation metrics included accuracy, sensitivity, AUC, fitness value, and the number of selected features. The same classifier and dataset splits were applied to each method to ensure consistent benchmarking.

The proposed approach introduces a hybrid architecture combining SSDAE and MLP for feature representation, integrated with an enhanced optimization framework based on a modified Hiking Optimization Algorithm. The use of Dynamic Opposite Learning increases population diversity, while the Double Attractors mechanism improves convergence toward better feature subsets. These enhancements were specifically designed to address the high dimensionality and noise in rs-fMRI data and contribute to improved classification outcomes across the datasets.

The results, summarized in Tables 1–3, demonstrate that MHOA consistently achieved competitive performance compared to the other algorithms in most evaluation metrics.

**Table 1:** Results of the Dataset-1.

**Measure**		**MHOA**	**HOA**	**SMA**	**AROA**	**HHO**	**GWCA**	**GWO**
Accuracy	Mean	**0.7019**	0.6779	0.6635	0.6442	0.6490	0.6587	0.6298
	StDev	0.0272	**0.0068**	0.0136	0.0136	0.0204	**0.0068**	0.0476
	Best	**0.7212**	0.6827	0.6731	0.6538	0.6635	0.6635	0.6635
	Worst	**0.6827**	0.6731	0.6538	0.6346	0.6346	0.6538	0.5962
Sensitivity	Mean	**0.7400**	0.7200	0.6700	0.7000	0.6600	0.7200	0.6800
	StDev	0.0566	0.0283	0.0424	**0.0000**	0.0283	**0.0000**	0.0283
	Best	**0.7800**	0.7400	0.7000	0.7000	0.6800	0.7200	0.7000
	Worst	0.7000	0.7000	0.6400	0.7000	0.6400	**0.7200**	0.6600
AUC	Mean	**0.7668**	0.7318	0.7161	0.6678	0.7239	0.7092	0.7100
	StDev	0.0111	**0.0034**	0.0327	0.0079	0.0259	0.0164	0.0352
	Best	**0.7746**	0.7342	0.7392	0.6733	0.7422	0.7207	0.7349
	Worst	**0.7590**	0.7294	0.6930	0.6622	0.7056	0.6976	0.6851
Fitness value	Mean	0.2777	0.2732	0.3027	0.3102	**0.2689**	0.3664	0.2698
	StDev	0.0073	0.0048	0.0088	0.0831	0.0281	**0.0040**	0.0253
	Best	0.2725	0.2698	0.2964	0.2514	**0.2490**	0.3635	0.2519
	Worst	0.2828	**0.2766**	0.3089	0.3689	0.2887	0.3692	0.2876
Features no.	Best	**65.00**	102.00	87.00	262.00	92.00	415.00	116.00
	StDev	16.47	**7.78**	66.26	167.58	94.75	24.04	31.82
	Mean	**76.00**	107.50	134.50	380.50	159.00	432.00	138.50

Table 1 lists the numerical results for DATASET-1. In the table, the accuracy results demonstrated that MHOA achieved the highest mean accuracy, followed closely by HOA and SMA. The stability of MHOA was slightly lower than HOA, as indicated by its higher standard deviation. However, its best and worst accuracy values remained superior to those of the other methods. SMA exhibited moderate performance, with AROA and HHO showing lower mean values. GWO achieved the lowest accuracy.

In terms of sensitivity, MHOA ranked first, achieving the highest mean value. HOA and GWCA followed with comparable mean sensitivity scores, while AROA and GWO were positioned in the middle. HHO showed the lowest mean sensitivity. The standard deviation analysis revealed that AROA and GWCA had the most stable results, while MHOA exhibited intermediate stability. The best sensitivity values confirmed MHOA's advantage, as it reached the highest observed value, while the worst results indicated that GWCA achieved the highest stability, followed by MHOA and HOA.

Regarding the AUC metric, MHOA achieved the highest mean, indicating superior overall classification performance. HOA and HHO also showed competitive results, with SMA ranking slightly lower. AROA exhibited the lowest mean AUC. Standard deviation values indicated that HOA maintained the most stable performance, whereas SMA and GWO showed greater variability. The best and worst AUC values showed MHOA's effectiveness, as it outperformed the compared methods.

For the fitness value, HHO exhibited the best mean. HOA and GWO followed closely, with MHOA ranking slightly lower. However, the standard deviation results indicated that MHOA and HOA provided more stable optimization performance than HHO and AROA, which had higher variance. The best and worst fitness values showed that HHO maintained a strong optimization capability, while GWCA had the highest worst fitness value.

**Figure 2:**
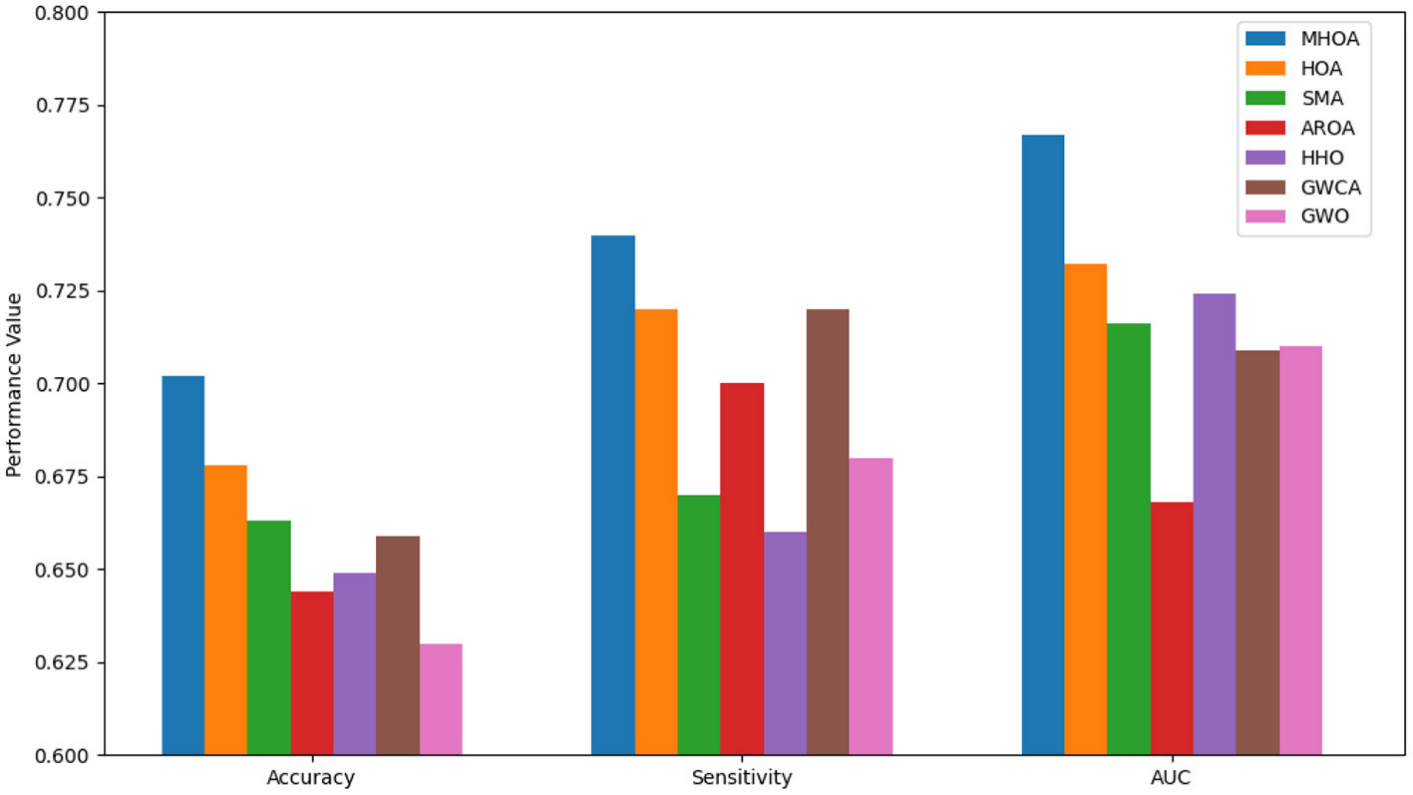
Results of accuracy, sensitivity, and AUC for Dataset-1.

The FS results revealed that MHOA achieved the lowest number of selected features in the Best-case scenario. SMA and HOA followed, while GWCA selected the highest number of features. The standard deviation results indicated that HOA had the most stable FS followed by MHOA, while AROA and HHO exhibited greater variability. The mean values confirmed that MHOA consistently selected fewer features than the other methods and demonstrated its efficacy in reducing dimensionality while preserving classification performance. Figure 2 presents a comparison of the algorithms' performance on Dataset-1 across Accuracy, Sensitivity, and AUC metrics.

As shown in Figure 2, the MHOA algorithm consistently outperformed other algorithms across all three key metrics: accuracy, sensitivity, and AUC. Notably, MHOA achieved the highest average accuracy of 0.7019, indicating improved classification capability. These results validate the effectiveness of combining SSDAE-based feature extraction with the modified HOA in capturing discriminative features from rs-fMRI data.

Table 2 presents the results of the DATASET-2. In the table, MHOA achieved the highest mean accuracy, followed closely by AROA, HHO, and GWCA, which exhibited similar performance. The standard deviation values showed that HOA and GWO exhibited the most stable results. MHOA outperformed other methods in the best and worst accuracy values. SMA, AROA, and HHO exhibited moderate performance, while HOA and GWO had lower rankings.

**Table 2:** Results of the Dataset-2.

**Measure**		**MHOA**	**HOA**	**SMA**	**AROA**	**HHO**	**GWCA**	**GWO**
Accuracy	Mean	**0.6635**	0.6442	0.6442	0.6490	0.6490	0.6490	0.6442
	StDev	0.0136	**0.0000**	0.0136	0.0068	0.0068	0.0068	**0.0000**
	Best	**0.6731**	0.6442	0.6538	0.6538	0.6538	0.6538	0.6442
	Worst	**0.6538**	0.6442	0.6346	0.6442	0.6442	0.6442	0.6442
Sensitivity	Mean	0.7300	0.7100	**0.7400**	0.7000	0.7300	0.7200	0.7200
	StDev	0.0424	0.0141	0.0283	**0.0000**	0.0141	**0.0000**	**0.0000**
	best	**0.7600**	0.7200	**0.7600**	0.7000	0.7400	0.7200	0.7200
	Worst	0.7000	0.7000	**0.7200**	0.7000	**0.7200**	**0.7200**	**0.7200**
AUC	Mean	**0.6931**	0.6873	0.6711	0.6839	0.6794	0.6781	0.6839
	StDev	0.0076	0.0064	0.0113	0.0060	0.0028	**0.0009**	0.0058
	Best	**0.6985**	0.6919	0.6791	0.6881	0.6814	0.6787	0.6880
	Worst	**0.6878**	0.6828	0.6631	0.6796	0.6774	0.6774	0.6798
Fitness value	Mean	**0.1658**	0.3484	0.2846	0.2626	0.2877	0.3482	0.3187
	StDev	**0.0036**	0.0047	0.0089	0.0176	0.0201	0.0065	0.0230
	Best	**0.1633**	0.3450	0.2784	0.2501	0.2735	0.3436	0.3025
	Worst	**0.1683**	0.3517	0.2909	0.2751	0.3019	0.3528	0.3349
Features no.	Best	16.00	74.00	**10.00**	41.00	48.00	58.00	51.00
	StDev	3.54	1.41	14.85	**0.71**	7.78	5.66	10.61
	Mean	**18.50**	75.00	20.50	41.50	53.50	62.00	58.50

In terms of sensitivity, SMA achieved the highest mean value, with MHOA and HHO ranking closely behind. HOA and AROA exhibited the lowest sensitivity. Standard deviation values showed that AROA, GWCA, and GWO were the most stable. The Best-case confirmed MHOA's strength, while the worst value demonstrated that SMA, HHO, GWCA, and GWO showed similar results, followed by MHOA.

Regarding the AUC metric, MHOA ranked highest in mean performance and demonstrated its superior classification capability. HOA followed closely, while SMA recorded the lowest mean AUC. Standard deviation results indicated that GWCA maintained the most stable AUC performance, whereas SMA showed greater variability. The best and worst values confirmed MHOA's consistently high performance across various scenarios.

For the fitness value, MHOA exhibited the best mean. AROA followed, while HOA recorded the highest mean value. The standard deviation analysis showed that MHOA had the most stable fitness value, whereas GWO and HHO exhibited greater variability. The best and worst fitness values further confirmed that MHOA maintained strong optimization capability, whereas HOA and GWCA showed inconsistent performance.

**Figure 3:**
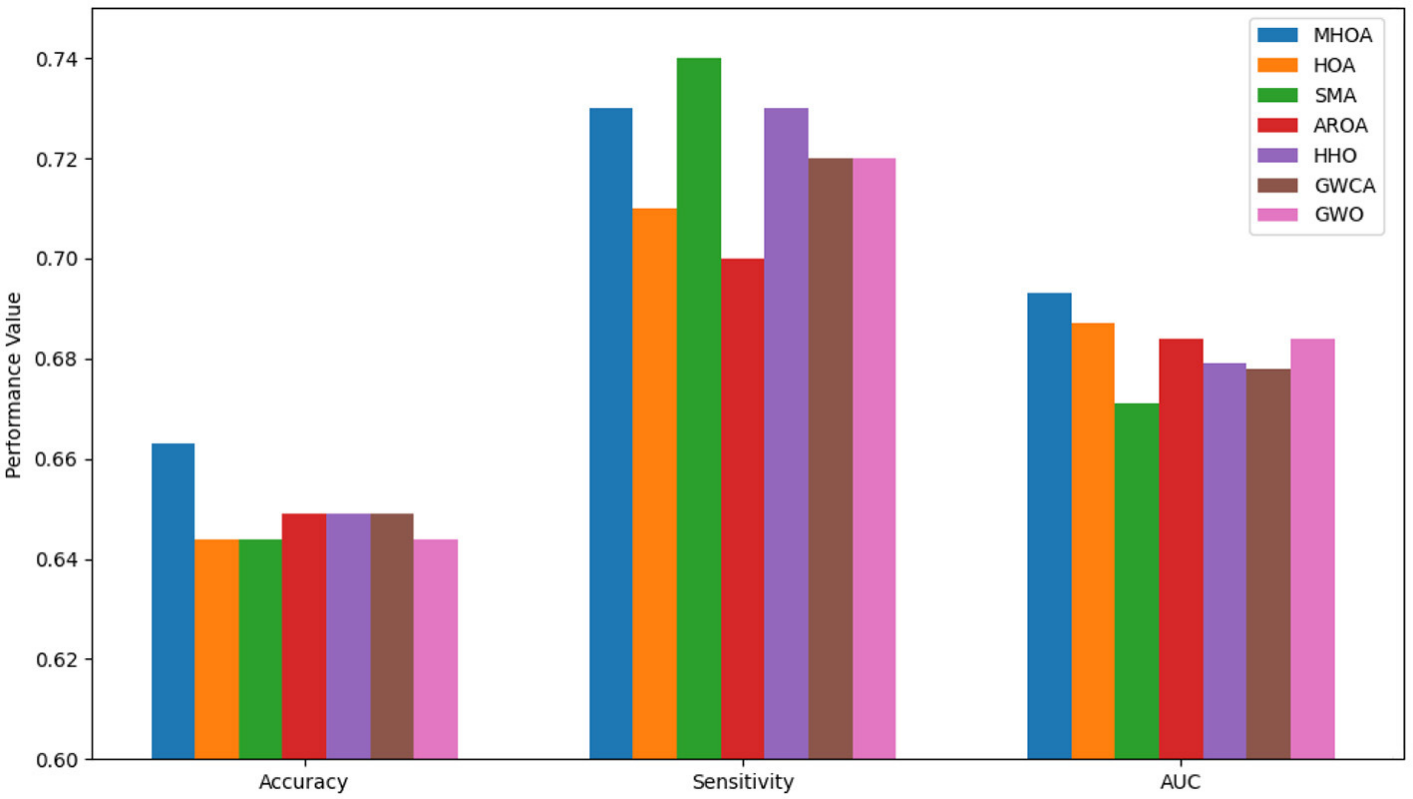
Results of accuracy, sensitivity, and AUC for Dataset-2.

The FS results revealed that SMA achieved the lowest number of selected features in the best case. MHOA followed closely, while HOA selected the highest number of features. The standard deviation values indicated that AROA maintained the most stable FS, followed by HOA and MHOA, while SMA and GWO exhibited greater variability. The mean values confirmed that MHOA consistently selected fewer features than most methods and proved effective in dimensionality reduction while maintaining classification accuracy. Figure 3 presents a comparison of the algorithms' performance on Dataset-2 across Accuracy, Sensitivity, and AUC metrics.

In Figure 3, the proposed MHOA model outperforms all competing methods in terms of accuracy and AUC, demonstrating improved classification capability. Although the SMA algorithm achieved slightly higher sensitivity, MHOA exhibits a strong balance between sensitivity and accuracy, which is crucial in minimizing both false negatives and false positives in ASD detection. The GWCA and GWO models showed relatively stable sensitivity but did not match the overall classification performance of MHOA.

Table 3 presents the results and shows that MHOA achieved the highest mean accuracy. AROA and GWO followed closely, while HOA and SMA ranked lower. Standard deviation values indicated that AROA and GWO exhibited stable performance, whereas HOA and SMA showed greater variability. MHOA maintained its dominance in best and worst accuracy values, reinforcing its robustness.

**Table 3:** Results of the Dataset-3.

**Measure**		**MHOA**	**HOA**	**SMA**	**AROA**	**HHO**	**GWCA**	**GWO**
Accuracy	Mean	**0.8382**	0.8261	0.8164	0.8333	0.8309	0.8285	0.8333
	StDev	0.0034	0.0137	0.0137	**0.0034**	0.0068	0.0171	**0.0034**
	Best	**0.8406**	0.8357	0.8261	0.8357	0.8357	**0.8406**	0.8357
	Worst	**0.8357**	0.8164	0.8068	0.8309	0.8261	0.8164	0.8309
Sensitivity	Mean	**0.8257**	0.7936	0.7890	0.8165	0.8211	0.8028	0.8211
	StDev	**0.0000**	0.0195	0.0130	**0.0000**	0.0065	0.0324	0.0065
	best	**0.8257**	0.8073	0.7982	0.8165	**0.8257**	**0.8257**	**0.8257**
	Worst	**0.8257**	0.7798	0.7798	0.8165	0.8165	0.7798	0.8165
AUC	Mean	**0.9099**	0.8980	0.9006	0.9065	0.9085	0.9008	0.9065
	StDev	0.0013	0.0018	0.0045	**0.0010**	0.0028	0.0059	0.0029
	Best	**0.9108**	0.8993	0.9038	0.9072	0.9105	0.9050	0.9086
	Worst	**0.9091**	0.8967	0.8974	0.9058	0.9065	0.8966	0.9045
Fitness value	Mean	0.2100	0.2048	**0.1826**	0.2382	0.2681	0.2029	0.2358
	StDev	0.0257	0.0084	0.0021	0.0274	**0.0018**	0.0034	0.0300
	Best	0.1919	0.1989	**0.1811**	0.2188	0.2668	0.2005	0.2146
	Worst	0.2282	0.2108	**0.1841**	0.2576	0.2694	0.2053	0.2570
Features no.	Best	35.00	**15.00**	20.00	57.00	73.00	22.00	36.00
	StDev	16.26	9.90	12.12	**6.36**	13.44	15.56	29.70
	Mean	46.50	22.00	**21.50**	61.50	82.50	33.00	57.00

Regarding sensitivity, MHOA ranked the highest with the best mean performance. HHO and GWO followed closely behind. HOA showed the lowest sensitivity and indicated its weakness in detecting positive instances. Standard deviation results indicated that MHOA and AROA were the most stable in sensitivity, while SMA, HOA, and GWCA exhibited higher variability. The best-case values demonstrated that MHOA consistently performed well in identifying positive instances, while HOA's worst-case results showed significant variability.

For AUC, MHOA outperformed all other algorithms in mean performance and demonstrated clear class differentiation capability. HHO and GWO ranked next, while HOA performed the weakest in AUC. Standard deviation analysis revealed that AROA exhibited the most stability in AUC performance, while SMA and GWCA showed higher fluctuations. The best and worst values confirmed that MHOA maintained good performance across different scenarios and indicated its reliability in class separation.

In terms of fitness value, SMA achieved the lowest mean. HOA and GWCA followed, while HHO recorded the highest mean. Standard deviation results showed that HHO was the most stable in optimization performance, while AROA and GWO exhibited higher variability. The best and worst values further supported SMA's optimization efficiency, while MHOA exhibited competitive performance with relatively stable optimization. HHO and AROA displayed less stability in converging to optimal solutions.

**Figure 4:**
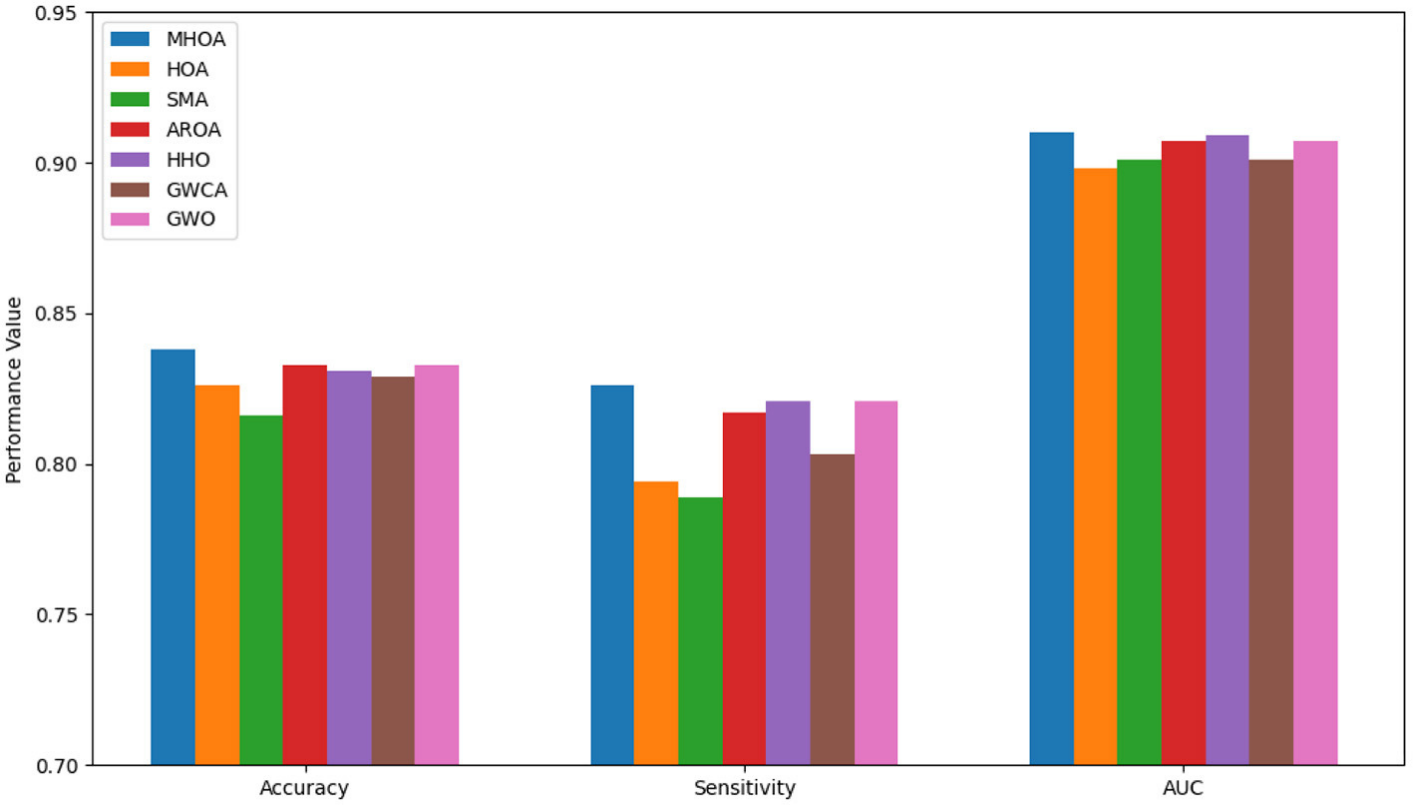
Results of accuracy, sensitivity, and AUC for Dataset-3.

Regarding FS, HOA selected the fewest features in the best-case scenario. SMA followed closely behind, while HHO selected the highest number of features. MHOA demonstrated a balanced approach by selecting a moderate number of features, maintaining a trade-off between dimensionality reduction and classification performance. Standard deviation values revealed that AROA exhibited the most stable FS, while GWO showed the greatest variability. The mean values confirmed that HOA and SMA consistently selected fewer features, while MHOA maintained a competitive balance between FS and model performance. Figure 4 presents a comparison of the algorithms' performance on Dataset-3 across Accuracy, Sensitivity, and AUC metrics.

As shown in Figure 4, the MHOA algorithm achieved the best results across all metrics. It ranked first in accuracy, followed by AROA, GWO, and HHO. In sensitivity, MHOA again led, while HHO, GWO, and AROA showed similar outcomes. For AUC, MHOA maintained the top position, with HHO, AROA, and GWO close behind. GWCA and HOA showed lower sensitivity and AUC values, and SMA ranked lowest in accuracy and sensitivity.

These observed performance rankings are further supported by the results of the Friedman test, a non-parametric statistical method commonly used to detect differences across multiple conditions when the data does not follow normal distribution assumptions. It is particularly useful in model comparison as it accounts for the ordinal nature of the data and dependencies between repeated measures. The results of the Friedman test, as shown in Table 4, reveal considerable variability in performance across the models. MHOA consistently ranks highest in accuracy, AUC, and sensitivity and shows better overall effectiveness. In contrast, GWO ranks lowest in accuracy and reflects relatively lower performance in this context. Models such as HOA, SMA, AROA, HHO, and GWCA demonstrate intermediate performance. These findings highlight differences among the models, with MHOA showing the most consistent results.

**Table 4:** Results of the Friedman test.

**Measure**	**MHOA**	**HOA**	**SMA**	**AROA**	**HHO**	**GWCA**	**GWO**
Accuracy	**7.00**	3.33	2.67	4.17	4.00	4.00	2.83
AUC	**7.00**	4.33	2.50	3.33	4.67	2.17	4.00
Sensitivity	**6.50**	3.17	3.33	3.00	4.00	4.00	4.00

To further evaluate the performance of the developed MHOA model to detect ASD, we compared it with the results obtained in Liu et al. (2024). Since this work uses the same strategy to split ABIDE I dataset. The technique used in Liu et al. (2024) is named MADE-for-ASD, and its accuracy for CC, AAL, and EZ is 73.42%, 71.20%, and 68.74%, respectively. However, our developed MHOA based on the SSDAE model has accuracy 66.35%, 70.19%, and 83.82% for EZ, AAL, and CC, respectively. So, MADE-for-ASD is better than MHOA at EZ and AAL, whereas MHOA is better according to the results of CC split (dataset-3). In addition, the average accuracy of MADE-for-ASD and our MHOA model overall the three datasets is 71.12% and 73.45%, respectively. This indicates the high ability of the developed model to detect ASD.

In general, MHOA consistently achieved the highest accuracy and AUC across datasets and demonstrated strong classification performance. Sensitivity results confirmed its ability to identify positive instances. Its optimization performance remained competitive with relatively stable fitness values. FS analysis indicated that MHOA maintained a balance between dimensionality reduction and model effectiveness and demonstrating its reliability across diverse evaluation criteria. However, its stability in some metrics, such as fitness value and sensitivity, was lower than that of certain methods and suggesting potential improvements in robustness under varying conditions.

## 6 Conclusion and future works

This paper presents a novel DL model integrated with a modified version of the HOA for detecting ASD from rs-fMRI data. The proposed model enhances the accuracy of ASD detection, potentially improving early intervention strategies for individuals who may otherwise be missed by traditional methods. By combining the SSDAE and MLP, the model effectively extracts relevant features, while the enhanced HOA, utilizing dynamic opposite-based learning and double attractors, optimizes FS. The developed model demonstrates promising results, with an average accuracy of 0.735, sensitivity of 0.765, and AUC of 0.790 across various datasets, showing the potential of DL and the MHOA algorithm in automated ASD detection.

Despite the promising results, several challenges remain. The biological heterogeneity of ASD, along with variations in imaging protocols and preprocessing steps, introduces potential limitations that could affect the generalizability and reproducibility of the model. Future work could focus on addressing these challenges by incorporating multi-site datasets with consistent preprocessing pipelines to enhance the model's robustness and external validity. Additionally, the model's interpretability remains a key consideration, as understanding the decision-making process of DL models is crucial for clinical adoption. Efforts toward developing explainable AI techniques could be integrated to provide more transparent insights into the detected features and their relevance to ASD. Moreover, future research should explore the application of this approach to other neurodevelopmental disorders that exhibit overlapping symptoms with ASD, such as ADHD and intellectual disabilities. Expanding the model's applicability across a broader spectrum of neurodevelopmental conditions could facilitate the development of more generalized and efficient diagnostic tools. Furthermore, incorporating longitudinal data and examining the model's performance over time could provide deeper insights into the progression of ASD and its early detection.

In conclusion, this study lays the groundwork for a more effective, DL-based diagnostic tool for ASD, offering a promising direction for early detection and intervention. However, further refinements and validations in diverse clinical settings are necessary to ensure the model's practical applicability in real-world healthcare environments.

## Data Availability

Publicly available datasets were analyzed in this study. This data can be found here: https://www.nature.com/articles/s41598-022-09821-6.
